# Prolactin Blood Test Compliance and Documentation in Adult Inpatient Settings

**DOI:** 10.1192/bjo.2026.11360

**Published:** 2026-06-30

**Authors:** Neelam Choudhary, Amitav Narula

**Affiliations:** Black Country Healthcare NHS Foundation Trust, Dudley, United Kingdom

## Abstract

**Aims:**

Various psychotropic medications have an impact on serum prolactin levels. Measurement of prolactin is therefore an important step in patient management. Clear documentation of prolactin levels and subsequent action plan will help to minimise the risk of missed prolactin results.

**Methods:**

Screening of clinical records in an acute inpatient ward was undertaken to assess compliance with prolactin blood test measurement and recording of subsequent action plan. It was found that 55% of the inpatients had this completed. This led to a quality improvement project where a root cause analysis was undertaken. As part of the process survey wascompleted by medical teams caring for the inpatients to identify barriers to testing and documentation.

As per survey barriers to prolactin testing included human error such as forgetting to add prolactin to blood form, infrequent phlebotomy staff visits, lack of awareness of monitoring requirements and unclear responsibility for requesting tests. On the other hand, limited access to prolactin management guidelines, lack of standardized documentation template and time constraints during ward review were barriers to documentation of action plan. Based on these findings, the following interventions were implemented. Written reminders were placed on wards, meeting held with phlebotomy services to clarify visit schedule and collaboration withelectronic records team to add a dedicated section on capturing blood test and subsequent action plan within clinical records.

**Results:**

Following the introduction of written reminders on the ward, a modest improvement was observed in compliance with prolactin blood test monitoring. Relatively bigger improvement was noted in the documentation of associated action plans.

Electronic records team is set to make changes to clinical records by adding a section dedicated to capturing blood results and its action plan by January 2026. This intervention is anticipated to lead to further improvements in both monitoring and documentation compliance.

**Conclusion:**

In summary the project identified various barriers contributing to poor prolactin monitoring and documentation of subsequent action plan in inpatient wards. Targeted intervention of written reminders led to modest improvement. Incorporating prolactinmonitoring within electronic clinical record from January 2026 is anticipated to achieve greater improvement.

Recommendations: Trust to consider blood stamps to improve compliance with complete set of admission bloods.

